# Effect of Light Flashes vs Sham Therapy During Sleep With Adjunct Cognitive Behavioral Therapy on Sleep Quality Among Adolescents: A Randomized Clinical Trial

**DOI:** 10.1001/jamanetworkopen.2019.11944

**Published:** 2019-09-25

**Authors:** Katherine A. Kaplan, Meital Mashash, Rayma Williams, Holly Batchelder, Lolly Starr-Glass, Jamie M. Zeitzer

**Affiliations:** 1 Stanford Center for Sleep Sciences and Medicine, Department of Psychiatry and Behavioral Sciences, Stanford University, Stanford, California; 2 Palo Alto University, Palo Alto, California

## Abstract

**Importance:**

Owing to biological, behavioral, and societal factors, sleep duration in teenagers is often severely truncated, leading to pervasive sleep deprivation.

**Objective:**

To determine whether a novel intervention, using both light exposure during sleep and cognitive behavioral therapy (CBT), would increase total sleep time in teenagers by enabling them to go to sleep earlier than usual.

**Design, Setting, and Participants:**

This double-blind, placebo-controlled, randomized clinical trial, conducted between November 1, 2013, and May 31, 2016, among 102 adolescents enrolled full-time in grades 9 to 12, who expressed difficulty going to bed earlier and waking up early enough, was composed of 2 phases. In phase 1, participants were assigned to receive either 3 weeks of light or sham therapy and were asked to try to go to sleep earlier. In phase 2, participants received 4 brief CBT sessions in addition to a modified light or sham therapy. All analyses were performed on an intent-to-treat basis.

**Interventions:**

Light therapy consisted of receiving a 3-millisecond light flash every 20 seconds during the final 3 hours of sleep (phase 1) or final 2 hours of sleep (phase 2). Sham therapy used an identical device, but delivered 1 minute of light pulses (appearing in 20-second intervals, for a total of 3 pulses) per hour during the final 3 hours of sleep (phase 1) or 2 hours of sleep (phase 2). Light therapy occurred every night during the 4-week intervention. Cognitive behavioral therapy consisted of four 50-minute in-person sessions once per week.

**Main Outcomes and Measures:**

Primary outcome measures included diary-based sleep times, momentary ratings of evening sleepiness, and subjective measures of sleepiness and sleep quality.

**Results:**

Among the 102 participants (54 female [52.9%]; mean [SD] age, 15.6 [1.1] years), 72 were enrolled in phase 1 and 30 were enrolled in phase 2. Mixed-effects models revealed that light therapy alone was inadequate in changing the timing of sleep. However, compared with sham therapy plus CBT alone, light therapy plus CBT significantly moved sleep onset a mean (SD) of 50.1 (27.5) minutes earlier and increased nightly total sleep time by a mean (SD) of 43.3 (35.0) minutes. Light therapy plus CBT also resulted in a 7-fold greater increase in bedtime compliance than that observed among participants receiving sham plus CBT (mean [SD], 2.21 [3.91] vs 0.29 [0.76]), as well as a mean 0.55-point increase in subjective evening sleepiness as compared with a mean 0.48-point decrease in participants receiving sham plus CBT as measured on a 7-point sleepiness scale.

**Conclusions and Relevance:**

This study found that light exposure during sleep, in combination with a brief, motivation-focused CBT intervention, was able to consistently move bedtimes earlier and increase total sleep time in teenagers. This type of passive light intervention in teenagers may lead to novel therapeutic applications.

**Trial Registration:**

ClinicalTrials.gov identifier: NCT01406691

## Key Points

**Question:**

Can adjustment of circadian timing through light flash therapy during sleep increase total sleep time in teenagers?

**Findings:**

In this double-blind, placebo-controlled, randomized clinical trial of 102 adolescents, light flash therapy alone was ineffective. When delivered in combination with a cognitive behavioral therapy meant to encourage an earlier bedtime, the combination of light flash therapy and cognitive behavioral therapy significantly and stably increased total sleep time by nearly 45 minutes per night.

**Meaning:**

Combination light flash therapy and cognitive behavioral therapy is an effective tool to increase sleep time and combat sleep loss in teenagers.

## Teaser

This randomized clinical trial examines whether a novel intervention, using both light exposure during sleep and cognitive behavioral therapy, increases total sleep time in teenagers by enabling them to go to sleep earlier than usual.

## Introduction

Insufficient sleep in adolescence is increasingly recognized as a public health concern, with implications for academic performance, health, safety, and psychological well-being. Across the previous 2 decades, sleep durations in adolescents have been declining.^1^ Nearly half of US teenagers report regular insufficient sleep and excessive daytime sleepiness, particularly on school days.^2,3^ In one sample of 100 middle-school and high-school students, 80% slept fewer than 8 hours on school nights, with 44% of the students reporting difficulty staying awake during school and 85% reporting using caffeine to combat daytime fatigue.^3^ The implications of insufficient sleep are far reaching. Associations have been established between sleep duration and teenagers’ grade point average,^4,5^ physical health,^6,7^ substance use and risk-taking behaviors,^8,9^ and psychological well-being.^10,11,12^

Multiple factors contribute to insufficient sleep in adolescence. Empirical data indicate that adolescents sleep less with age,^13,14^ even as the need for sleep remains constant or increases.^15^ This decrement in sleep is likely owing to a combination of later bedtimes, coupled with fixed, earlier school start times,^2,16^ which results in insufficient time for sleep. Later bedtimes are thought to be caused by a set of reinforcing influences: a naturally occurring delay in the timing of the circadian clock^16^ and increasing psychosocial activities at night (eg, work or homework, socialization, and use of electronics).^3,17^ These nocturnal activities can laten bedtime both directly and indirectly, owing to the concomitant evening light exposure that further delays the timing of the circadian clock.

Previous interventions have attempted to address this latening of bedtime using a combination of early morning phototherapy (to move the timing of the circadian clock to an earlier clock hour) and behavioral or education-based interventions (to target psychosocial and environmental factors), with mixed results. Classroom-based interventions, designed to improve sleep through education^18,19^ or through administration of in-class morning light therapy,^20^ have not been successful in changing weekday sleep durations. One trial using light therapy and cognitive behavioral therapy (CBT) improved the sleep of adolescents meeting criteria for delayed sleep phase disorder,^21^ but its applicability to a broader population of teenagers with sleep difficulties is unknown. Although the use of CBT for insomnia is a widely validated method,^22^ it is unlikely to be useful as a stand-alone treatment for delayed sleep, which is not equivalent to insomnia, as there are innate biological reasons (ie, delay in circadian timing) that would be difficult to overcome with behavioral efforts. Use of behavioral therapy to promote sleep at an earlier circadian phase (without shifting the clock to an earlier phase) may, in fact, lead to the induction of insomnia.^23^

Traditional bright light phototherapy in the morning is designed to offset the natural delay in circadian timing (and bedtime) by evoking an advance in the timing of the circadian clock (ie, events on subsequent days are set to an earlier time). In adolescents, exposure to bright light prior to their habitual wake time, and to a lesser degree after awakening, will advance the timing of the circadian clock.^24,25^ In theory, this exposure would realign bedtime to a time more amenable to getting sufficient sleep. Such a change in circadian timing, however, would be temporary and would need to occur each day to offset the naturally occurring biological delay associated with adolescence. Also, traditional light therapy often requires teenagers to awaken earlier than their habitual wake time, which engenders even greater sleep loss. However, one study has shown that the human circadian system can respond to sequences of extraordinarily brief, millisecond-length flashes of light while people are sleeping (flash therapy), and that such light does not disrupt sleep.^26^ The physiological circuit underlying the circadian responses to light flashes is not completely understood, but the ability of the neurons that project from the retina to the suprachiasmatic nucleus to continue their electrical response for several minutes after cessation of light^27^ is likely to be in part responsible for the integration of light flashes over time (temporal integration). Using flashes of light is a powerful tool with which one can manipulate the circadian system, as it is more than 2- to 3-fold stronger than continuous light.^28^ Thus, flash therapy delivered during the last hours of habitual sleep could set the human circadian pacemaker to an earlier hour, allowing teenagers to go to sleep earlier, and without the burden of having to wake earlier than usual.

Our study consisted of 2 phases. Phase 1 examined whether, independent of any behavioral adjustments, flash therapy during sleep could move sleep timing earlier and increase total sleep in teenagers. Phase 2 examined whether flash therapy in combination with brief CBT could move sleep timing earlier and increase total sleep time in teenagers. In both phases, participants had 1 week of ad libitum baseline sleep monitoring at home with a written diary followed by 4 weeks of treatment. The primary outcome measures were the timing of the onset of sleep and the total amount of sleep time.

## Methods

### Participants

**Figure 1.**
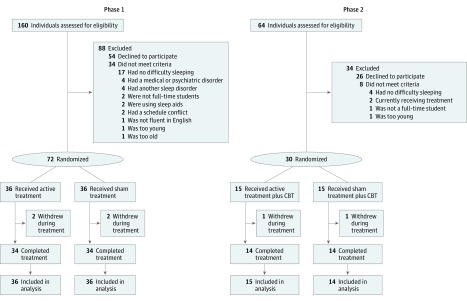
CONSORT Diagrams of Participant Flow in Phase 1 and Phase 2 CBT indicates cognitive behavioral therapy.

Phase 1 included 72 adolescents aged 14 to 18 years (recruited from November 1, 2013, to April 30, 2015), while phase 2 included 30 adolescents aged 14 to 18 years (recruited from October 1, 2015, to May 31, 2016) (Figure 1) (trial protocol is available in Supplement 1). Recruitment was accomplished via ongoing partnerships with local schools, pediatricians, and the Stanford Sleep Clinic, as well as posts to online bulletin boards and high school e-newsletters. Inclusion criteria were intentionally broad: students who were enrolled full-time in grades 9 to 12 and who expressed difficulty going to bed earlier and waking up early enough were eligible for the study. Exclusion criteria included taking medication specifically for sleep disorders (including over-the-counter medications or supplements such as melatonin), sleeping exclusively in the prone position, meeting criteria for bipolar disorder, meeting criteria for a sleep disorder other than delayed sleep phase disorder or insomnia disorder, or currently receiving treatment for a sleep disorder. All procedures were approved by the Stanford University Institutional Review Board and conformed to the principles laid out in the Declaration of Helsinki.^29^ Assent was obtained from the adolescent participants and consent was obtained from at least 1 parent of each participant. This study followed the Consolidated Standards of Reporting Trials (CONSORT) reporting guideline.

### Procedure

**Figure 2.**
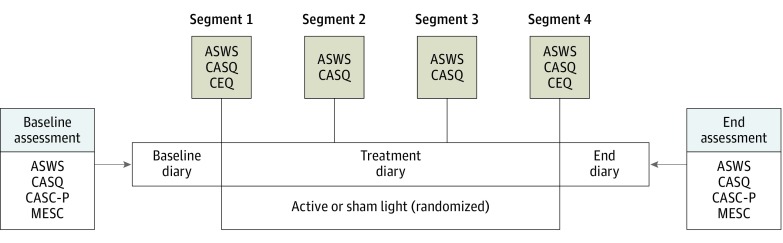
Protocol Diagram Timing of the different questionnaires (Adolescent Sleep-Wake Scale [ASWS], Cleveland Adolescent Sleepiness Questionnaire [CASQ], Child and Adolescent Sleep Checklist for parents [CASC-P], Credibility/Expectancy Questionnaire [CEQ], and Morningness-Eveningness Scale for Children [MESC]) given during the baseline and end assessments, as well as the ASWS and CASQ given during segments 1 through 4, which were given either over the telephone (phase 1) or in person (phase 2).

Study procedures are illustrated in Figure 2. In general, both phases followed the same pattern: 1 week of baseline followed by 4 weeks of intervention. Phase 1 had 2 groups: light alone (light) and sham light alone (sham). Phase 2 also had 2 groups: light plus CBT and sham light plus CBT. In each phase, after ensuring that eligibility criteria were met, participants were randomly assigned to 1 of the 2 groups. Block randomization (n = 15, to account for possible seasonality effects) was completed double-blind using a computer-generated random sequence. All participants were correctly told they would receive light at night and that the purpose of the investigation was to advance bedtimes to increase sleep duration.

Participants visited the laboratory with a parent for a baseline evaluation, followed by 1 week of ad libitum baseline sleep monitoring at home with a written sleep diary. At the end of this week, a technician visited the participant’s home to install the light device in the participant’s bedroom. Once the light was placed, participants were instructed to go to bed 1 hour earlier than their usual bedtime for the remainder of the study, to allow opportunity for the light to take effect. In phase 1, participants were called weekly to fill out questionnaires on sleepiness and sleep quality, and to report whether they noticed the light and whether the light interfered with their sleep. In phase 2, instead of weekly telephone calls, participants came to the laboratory for weekly therapy sessions (CBT, described below) during which the same questionnaires were completed. At the end of the 4-week intervention, participants returned to the laboratory with a parent to complete an end-of-treatment evaluation. Participants completed all portions of the study during the academic year and not during scheduled winter and spring breaks. If a teenager slept in 2 households, the light was placed in each household at approximately the same position relative to the bed.

### Measures

At a baseline visit, participants completed the following questionnaires: Morningness-Eveningness Scale for Children (morning vs evening preference),^30^ Adolescent Sleep-Wake Scale (subjective sleep quality),^31^ Cleveland Adolescent Sleepiness Questionnaire (daytime sleepiness),^32^ Credibility/Expectancy Questionnaire (participant belief in and expectation of results from intervention),^33^ and a single question inquiring about the reasons for sleep difficulty. During the course of the study, participants completed a sleep diary^34^ every morning, answered a question about sleepiness delivered via text message 2 hours prior to baseline bedtime every evening (Ecological Momentary Analysis),^35^ and repeated the Adolescent Sleep-Wake Scale and Cleveland Adolescent Sleepiness Questionnaire each week of the intervention and again at the end of treatment. The Credibility/Expectancy Questionnaire was also repeated at the end of treatment. At baseline and the end of treatment, a parent completed the Child and Adolescent Sleep Checklist for parents (parent’s impression of participant’s sleep).^36^

### Intervention

#### Flash Therapy

Participants were randomized to receive an active or sham light stimulus for the duration of the treatment. In the active condition (light), daily light therapy (ie, all days of the week) in the form of brief light pulses began 3 hours (in phase 1) or 2 hours (in phase 2) before the participant’s target weekday wake time. Approximately 4000 lux of broad-spectrum white light (approximately 200-600 lux at the cornea after eyelid filtration) was delivered in each 3-millisecond flash, occurring 20 seconds apart. Light was generated by a custom-designed xenon flash bulb (Moflash Signaling). The sham group received 1 minute of light pulses (appearing in 20-second intervals, for a total of 3 pulses) each hour; this dose is believed to be insufficient to shift the circadian system.^28^ The light timers for both groups were preprogrammed by one of us (J.M.Z.) who was not involved in assessment at any point. The placement of the experimental light was done to minimize the distance between the light and normal sleeping position and was measured for each participant.

#### CBT Intervention

Consistent with other brief behavioral treatments for sleep disturbances, the CBT intervention consisted of four 50-minute, in-person sessions once per week delivered by a licensed clinical psychologist or trained doctoral student (K.A.K., H.B., and L.S.-G.). The CBT intervention had several core components, including (1) education on the circadian system, the effect of light, and physiological processes; (2) information about the role of sleep in domains relevant to adolescents (eg, athletic performance, physical appearance, weight management, and academics); (3) sleep hygiene and stimulus control, well-established insomnia treatment components designed to improve sleep and associated contextual cues; and (4) activity scheduling to wake up earlier on weekends and anticipate obstacles.

Motivational interviewing and values-based clarification were central features of the CBT intervention. Given that motivational interviewing to promote behavior change may be strengthened by parent participation,^37^ teenagers and parents, together with the study therapist, set collaborative goals for the teenagers’ sleep at the baseline evaluation. To further promote change, teenagers were asked at the baseline evaluation to reflect the importance of sleep and their level of confidence and readiness to make sleep changes. Motivational interviewing was woven into each subsequent treatment session, including exploring ambivalence about change where resistance was met. At baseline, teenagers completed a worksheet on personal strivings,^38^ listing domains that were important to them (eg, “improve at softball,” “be happy,” and “get into college”). The treatment was subsequently tailored to adolescents’ individual goals, and the therapist could highlight relationships between adequate sleep and athletic performance, mood, or academic pursuits, as dictated by the adolescent.

### Statistical Analysis

Group differences in demographic variables were analyzed using *t* tests for continuous data and χ^2^ tests for categorical data. Differences in baseline measures were also evaluated by calculating the standardized mean difference (SMD); an SMD of 0.2 is considered small.^39^ All analyses were based on the intent-to-treat model. For investigating changes in sleep diary variables, Ecological Momentary Analysis, and weekly sleep questionnaires, linear mixed-effects models allowing for random intercepts were used. The differential effect of treatment was also expressed as Cohen *d*, calculated by dividing model-estimated difference in mean change by observed pooled SD at baseline.^40^ Mixed-effects models were run in R, version 3.1.2 (R Project for Statistical Computing), using lme4, version 1.1-7. Two-tailed *P* values were generated using Satterthwaite’s approximations^41^ for degrees of freedom in lmerTest, version 2.0-29. *P* < .05 was considered significant.

To examine variability across the intervention period, we calculated for each individual the proportion of days in which adolescents were compliant with prescribed bedtimes, defined by attempts to sleep at or before the target bedtime. As target bedtimes and wake times were collaboratively set in treatment sessions in phase 2, working toward an advance of 60 minutes, we further calculated a ratio of bedtime compliance relative to wake time compliance. For example, an individual who woke at the target wake time on 20 days and went to sleep at the target bedtime on 10 days would have a ratio of 0.5. This ratio would reflect compliance with sleep initiation (thought to be under circadian and self-motivated control) relative to compliance with sleep termination (more fixed through school start time). Ratios were log-transformed prior to statistical analyses.

## Results

### Sample Characteristics

Among the 102 participants (54 female [52.9%]; mean [SD] age, 15.6 [1.1] years), 72 were enrolled in phase 1 and 30 were enrolled in phase 2. Study flow is depicted in Figure 1. Groups within each phase were mostly similar (Table 1). There were no differences in age or sex between those who withdrew (phase 1, 4 of 72 [5.6%]; and phase 2, 2 of 30 [6.7%]) and those who completed the studies. There also were no significant differences in baseline measures between individuals who completed vs those who withdrew in terms of daytime sleepiness (mean [SD] Cleveland Adolescent Sleepiness Questionnaire score, 40.3 [9.1] vs 42.9 [13.7]; SMD = −0.27; *P* = .52), subjective sleep quality (mean [SD] Adolescent Sleep-Wake Scale score, 18.2 [2.8] vs 17.6 [3.5]; SMD = 0.21; *P* = .63), and parents’ impression of their teenager’s sleep (mean [SD] Child and Adolescent Sleep Checklist for parents score, 16.9 [5.8] vs 17.2 [11.1]; SMD = −0.046; *P* = .95). There was no singular reason teenagers gave for being unable to go to sleep earlier than their current bedtime. One teenager in phase 2, randomized to the sham plus CBT group, was excluded from analyses after reporting that an illness during both the baseline and posttreatment periods yielded unrepresentative sleep.

**Table 1.** Demographic and Clinical Information for Study Samples at Baseline

Characteristic	Phase 1			Phase 2		
	Light (n = 36)	Sham (n = 36)	*P* Value for Statistical Difference	Light Plus CBT (n = 15)	Sham Plus CBT (n = 15)	*P* Value for Statistical Difference
Age, mean (SD), y	15.7 (1.6)	15.4 (1.1)	.29	15.6 (1.2)	15.7 (1.4)	.89
Sex, Female, No. (%)	21 (58.3)	18 (50.0)	.64	7 (46.7)	8 (53.3)	>.99
Race, No. (%)						
Asian	7 (19.4)	2 (5.6)	.21	6 (40.0)	5 (33.3)	.53
Black	0	1 (2.8)		0	0	
White	20 (55.6)	25 (69.4)		4 (26.7)	7 (46.7)	
>1 Race	3 (8.3)	5 (13.9)		5 (33.3)	2 (13.3)	
Unknown or declined	6 (16.7)	3 (8.3)		0	1 (6.7)	
Ethnicity, Hispanic, No. (%)	8 (22.2)	1 (2.8)	.01	1 (6.7)	0	>.99
MESC score, mean (SD)	22.81 (4.04)	21.64 (3.59)	.20	21.93 (3.20)	21.60 (5.70)	.84

Abbreviations: CBT, cognitive behavioral therapy; MESC, Morningness-Eveningness Scale for Children.

### Sleep Diary Outcomes

In phase 1, application of the light therapy alone was not associated with a change in sleep timing (Table 2). Sleep efficiency (ie, total sleep time divided by time in bed) improved 3% in the light group from baseline to the end of treatment (mean [SD], 89% [8.5%] vs 92% [8.9%]; *P* < .001), which was different from the 1% change in the sham group (mean [SD], 92% [5.0%] vs 93% [4.5%]; *P* = .049), although groups differed on this variable by 3% at baseline (mean [SD]: light group, 89% [8.5%] vs sham group, 92% [5.0%]; *P* = .04) (Table 2). In phase 1, however, adolescents in both groups were often not compliant with instructions to go to bed 1 hour earlier (111 of 896 days’ compliance [12.4%] in light group vs 74 of 931 days’ compliance [7.9%] in sham), resulting in considerable bedtime variability.

**Table 2.** Phase 1 and Phase 2 Outcomes by Randomization Condition at Baseline (Week 1) and End-Treatment (Week 5)^a^

Outcome	Phase 1					Phase 2				
	Mean (SD)		Cohen *d*		Mean (SD)			Cohen *d*	
	Light (n = 36)	Sham (n = 36)	Time	Time × Treatment	Light Plus CBT (n = 15)		Sham Plus CBT (n = 14)	Time	Time × Treatment
Sleep diary									
Sleep onset latency, min									
Baseline	35.4 (45.2)	19.4 (19.6)	−0.09	−0.14	19.9 (18.7)		17.1 (8.12)	−0.48^b^	−0.19
End of treatment	26.4 (49.6)	16.3 (14.7)			11.3 (6.8)		10.3 (5.6)		
Sleep onset time, h:min									
Baseline	24:16 (1:05)	24:11 (0:59)	−0.28^c^	−0.22	24:21 (0:47)		23:52 (0:35)	−0.51^d^	−0.61^d^
End of treatment	24:14 (1:01)	23:49 (1:01)			23:31 (0:47)		23:30 (0:55)		
Total sleep time, min									
Baseline	445.0 (60.5)	452.0 (48.0)	0.29	0.02	438.9 (30.0)		462.8 (43.3)	0.37	0.78^c^
End of treatment	460.0 (67.3)	465.2 (53.8)			482.1 (37.1)		474.8 (35.4)		
Time in bed, min									
Baseline	501.5 (61.9)	490.3 (54.0)	0.26	−0.20	481.2 (41.8)		497.4 (44.7)	0.13	0.50
End of treatment	501.8 (57.3)	502.7 (59.4)			506.5 (36.7)		501.9 (35.6)		
Sleep efficiency, %									
Baseline	89 (8.5)	92 (5.0)	0.07	0.25^c^	91 (4.9)		93 (3.0)	0.51^d^	0.39
End of treatment	92 (8.9)	93 (4.5)			95 (2.5)		95 (2.7)		
Sleep initiation, h:min									
Baseline	23:41 (1:08)	23:52 (1:02)	−0.27^d^	0.35^c^	00:01 (0:55)		23:35 (0:37)	−0.32^c^	−0.49^c^
End of treatment	23:47 (1:05)	23:33 (1:01)			23:19 (0:48)		23:20 (0:54)		
Out of bed, h:min									
Baseline	8:04 (0:41)	8:02 (0:48)	−0.03	0.16	8:02 (0:34)		7:51 (0:35)	−0.26	−0.09
End of treatment	8:09 (0:47)	7:58 (0:46)			7:46 (0:38)		7:42 (0:52)		
Sleep quality score^e^									
Baseline	3.3 (0.5)	3.4 (0.5)	0.26	−0.10	3.4 (0.6)		3.6 (0.7)	0.42^d^	0.30
End of treatment	3.4 (0.6)	3.5 (0.5)			3.9 (0.6)		3.8 (0.5)		
Ecological momentary assessment									
Evening sleepiness rating^f^									
Baseline	3.6 (0.9)	3.1 (1.1)	0.36^c^	0.03	3.4 (1.1)		3.6 (0.9)	−0.32	0.74^c^
End of treatment	4.0 (1.2)	3.4 (1.3)			3.9 (1.1)		3.1 (1.0)		
Questionnaire scores									
CASQ									
Baseline	43.5 (8.2)	41.9 (10.0)	−0.82^b^	−0.07	37.2 (8.7)		36.6 (8.1)	−0.71^c^	−0.28
End of treatment	35.3 (7.8)	34.4 (8.1)			29.6 (7.5)		29.9 (9.2)		
ASWS									
Baseline	17.6 (3.0)	17.9 (2.8)	1.33^b^	0.03	19.6 (2.4)		18.6 (3.0)	1.66^b^	−0.23
End of treatment	21.5 (2.6)	21.8 (3.8)			23.3 (2.7)		23.1 (4.0)		
CASC-P									
Baseline	17.2 (6.0)	16.0 (5.0)	−0.21	0	18.3 (7.2)		17.9 (8.6)	−0.29	−0.18
End of treatment	16.2 (5.4)	15.0 (5.2)			14.3 (9.5)		14.8 (8.0)		

Abbreviations: ASWS, Adolescent Sleep-Wake Scale; CASC-P, Child and Adolescent Sleep Checklist (parent completed); CASQ, Cleveland Adolescent Sleepiness Questionnaire; CBT, cognitive behavioral therapy.

^a^ The effect size (Cohen *d*) is shown for the effect of time (ie, between baseline and end of treatment in the sham group) and the interaction between time and treatment (ie, the differential effect of the treatment over time).

^b^ *P* < .001.

^c^ *P* < .05.

^d^ *P* < .01.

^e^ Scores range from 1 to 5, with a higher score indicating better sleep quality.

^f^ Scores range from 1 to 7, with a higher score indicating sleepiness.

**Figure 3.**
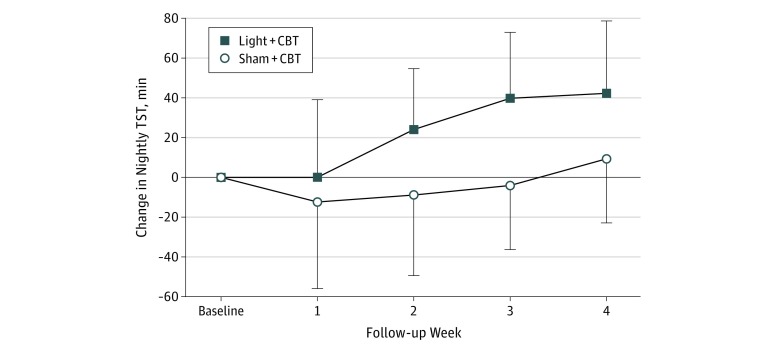
Change in Total Sleep Time (TST) Change in TST from ad libitum baseline through the 4 weeks of treatment in the light plus cognitive behavioral therapy (CBT) and sham plus CBT groups. By the end of treatment, those in the group receiving light plus CBT gained more than 40 minutes of extra sleep per night. Data were averaged within individuals by the week and then across individuals and are presented as mean (SD). Vertical lines indicate SD.

In phase 2, compared with the sham plus CBT group, the light plus CBT group showed greater improvement in sleep. The light plus CBT group had significantly earlier sleep onsets (mean [SD], 50.1 [27.5] minutes earlier), greater total sleep time (mean [SD], 43.3 [35.0] minutes longer), and earlier attempts to initiate sleep (mean [SD], 41.5 [32.5] minutes earlier) (Table 2, Figure 3). As an exploratory measure, we examined compliance in maintaining a regular bedtime, relativized to compliance with maintaining a regular wake time. In the sham plus CBT group, relative compliance was low (mean [SD], 0.29 [0.76]). In comparison, relative compliance in maintaining a regular bedtime in the light plus CBT group was 7-fold greater (mean [SD], 2.21 [3.91]; *t*_25_ = −3.13; *P* = .006). This finding was also reflected in significantly greater variability in bedtimes in the sham plus CBT group (11.5 minutes) compared with the light plus CBT group (21.5 minutes; *P* = .04, *F* test). Wake time variability did not differ between the groups (sham plus CBT, 24.3 minutes; and light plus CBT, 17.2 minutes; *P* = .25, *F* test).

### Ecological Momentary Analysis

In phase 1, both light and sham groups reported increased mean (SD) evening sleepiness ratings (light group: baseline, 3.62 [0.85]; and end of treatment, 4.01 [1.23]; and sham group: baseline, 3.14 [1.06]; and end of treatment, 3.39 [1.33]; *P* = .01) (Table 2). In phase 2, there was a significant time × treatment interaction present for evening sleepiness (*P* = .02) with increasing sleepiness in the light plus CBT group (+0.55; *P* = .07) and a nonsignificant decrease in sleepiness in the sham plus CBT group (−0.48; *P* = .15) during the evening (Table 2).

### Questionnaire Outcomes

All groups from both phases reported reductions in daytime sleepiness (Cleveland Adolescent Sleepiness Questionnaire) and increases in sleep quality (Adolescent Sleep-Wake Scale) (Table 2). There were no differences between treatments within phases. In phase 2, parents reported a 4-point reduction in their child’s sleep problems in the light plus CBT group (mean [SD] score: baseline, 18.3 [7.17]; and end of treatment, 14.3 [9.51]; *P* = .007) but not the sham plus CBT group (mean [SD] score: baseline, 17.9 [8.56]; and end of treatment, 14.8 [8.04]; *P* = .09) (Table 2, Child and Adolescent Sleep Checklist for parents).

### Light Placement and Tolerability

Light placement in participants’ bedrooms was a mean (SD) distance of 91.3 (62.2) cm (phase 1) and 111 (71.2) cm (phase 2) from participants’ pillows. There was no correlation between light placement and change in sleep onset time in either phase 1 (Pearson correlation, *r* = 0.11; *P* = .53) or phase 2 (Pearson correlation, *r* = 0.22; *P* = .46). In phase 1, more individuals in the light group relative to the sham group reported either noticing the light (29 vs 20; χ^2^_2_ = 5.92; *P* = .02; n = 68) or having their sleep disturbed by the light (19 vs 7; χ^2^_2_ = 8.97; *P* = .003; n = 68). To account for this disruption, the duration of the light exposure was reduced in phase 2. Subsequently, we found no differences in the number of teenagers in the light plus CBT group and in the sham plus CBT group who reported either seeing the light (14 vs 12; *P* > .99, Fisher exact test) or having their sleep disturbed by the light (6 vs 2; *P* = .22, Fisher exact test), although the low number of participants preclude us from concluding that there was no effect of the light on sleep.

### Treatment Equivalence

In phase 2, the intervention was rated favorably, with both groups demonstrating high mean (SD) Credibility/Expectancy Questionnaire scores at the end of segment 1 (light plus CBT group, 33.6 [6.1]; and sham plus CBT group, 34.7 [7.0]) and at the end of segment 4 (light plus CBT group, 36.0 [5.3]; and sham plus CBT group, 35.9 [6.7]). There were no differences in Credibility/Expectancy Questionnaire scores between the groups at the end of segment 1 (*t*_25_ = −0.41; *P* = .69) or at the end of segment 4 (*t*_23_ = 0.30; *P* = .98).

## Discussion

When given without supportive CBT, a sequence of 3-millisecond pulses of light delivered at the end of the nocturnal sleep episode was insufficient to bring about a change in sleep timing. When flash therapy was combined with a sleep-focused CBT intervention, however, we observed a robust advance in bed timing, decreases in time to fall asleep, and an increased nightly sleep duration of nearly 45 minutes. Without flash therapy, CBT alone did not significantly increase sleep and participant bedtimes were highly unstable, as teenagers were more than 7 times less compliant with earlier bed times as compared with their wake times. Our data are consistent with the theory that flash therapy advanced the timing of the circadian clock, enabling teenagers to initiate sleep at an earlier clock hour.^24,42^ The circadian clock elicits a strong wake-promoting signal in the hours immediately before normal bedtime.^43^ It is possible that adolescents in the sham plus CBT group attempted to initiate sleep when the circadian clock was signaling for wake, making it difficult to initiate sleep, thus creating lower compliance with the protocol and a potential to induce insomnia.^23^ In the light plus CBT group, circadian timing was likely moved earlier such that the adolescents in this group were not trying to go to sleep at a time during which the circadian clock was signaling for wake. This is supported by the Ecological Momentary Analysis sleepiness data: those in the light plus CBT group had greater sleepiness in the late evening, consistent with having their internal clock moved to an earlier time. Future experiments should include a direct measure of circadian timing to confirm this mechanism of action.

In phase 1, light flashes were both ineffective and somewhat disruptive to sleep. In response to this shortcoming, we decreased the duration of the flash sequences from 3 hours to 2 hours, which appeared to alleviate the negative effects of the light on sleep. Not only was there no greater notice or disruption of the light flashes, but teenagers also slept to the same clock hour in both conditions, indicating that the light flashes did not specifically disrupt the end of sleep (when the flashes were being delivered). It is also possible that shortening the flash sequence increased its effectiveness by reducing the likelihood that the flashes had either no effect or had a phase-delaying effect on circadian timing (ie, the light was more likely to elicit only circadian phase advances).^44^ Given that the light flashes in phase 1 (3 hours) and phase 2 (2 hours) are not identical, it would be important to examine whether 2 hours of light flashes were sufficient to change the time of sleep onset without the administration of adjunctive CBT. The calendar dates of therapy administration were not identical between phase 1 and phase 2, precluding direct comparison.

Future studies should include objective measurements of sleep (eg, actigraphy), given the possibility of reporting bias, as well as long-term monitoring of the light intervention. This latter improvement would be an important next step to determine ongoing acceptance of the light therapy and to determine whether the beneficial effects of light plus CBT could be maintained for months or years. The light sequence that we used (one 3-millisecond flash every 20 seconds for 2-3 hours) was selected based on early data.^42^ We have found more recently that a flash sequence occurring every 8 seconds is able to produce a change in circadian timing that is twice as large as the flash sequence used in the current study. If needed, bedtimes could be moved even earlier and more sleep could be obtained using an optimized light flash sequence, although convincing teenagers of the need for a further truncation of evening wake time could be difficult.

### Limitations

The light flashes in this study were delivered through a customized beacon. Although we did not observe any associations between the placement of the beacon in the adolescents’ rooms and any of our measures, it is possible that this is not an ideal form because the beacon is not commercially available and could affect other individuals sleeping in the same room (eg, siblings) who might not want to be affected by the light. Development of an alternative light delivery system (eg, eye mask based or pillow based) might be necessary in such conditions.

As previously mentioned, the use of objective sleep monitoring in a larger number of individuals will be critical in future studies of the effect of this intervention. Subjective sleep monitoring, actigraphic sleep monitoring, and polysomnographic sleep monitoring have some overlapping sources of variance but can be independent of one another, especially under conditions of poor sleep.^45,46,47^ There are shortcomings to each of these recording modalities, and the use of a combination of them may be necessary, especially in examination of the daytime sequelae of sleep deficits (eg, in academic performance), which is also an important area of follow-up.

## Conclusions

Despite the potential limitations of the light delivery mechanism, when combined with CBT, light flashes at the end of sleep enabled an earlier bedtime and increased nightly sleep by approximately 45 minutes in teenagers. This type of passive light intervention in teenagers may lead to novel therapeutic applications.

## Notes

Supplement 1. **Trial Protocol**

Supplement 2. **Data Sharing Statement**
