# Supplementary material for: Effect of Light Flashes vs Sham Therapy During Sleep With Adjunct Cognitive Behavioral Therapy on Sleep Quality Among Adolescents: A Randomized Clinical Trial
Source: JAMA Netw Open. 2019 Sep 25;2(9):e1911944. doi: 10.1001/jamanetworkopen.2019.11944 (PMC6763980; doi:10.1001/jamanetworkopen.2019.11944)
Supplement: Supplement 1. — Trial Protocol [file jamanetwopen-2-e1911944-s001.pdf]

Statistics.

The statistical and study design will be overseen by Dr. Booil Jo, a biostatistician at Stanford University. She is an expert in mixed model regression and experimental design of clinical trials, especially the handling of missing data. To examine the primary hypothesis concerning changes in the ASWS, we will analyze the longitudinal data from the baseline, during treatment, and end of treatment using a method widely known as mixed effects modeling or multilevel analysis. This strategy for modeling longitudinal data is known for better interpretability and to be more efficient than the univariate analytic methods such as ANOVA in terms of handling missing data. Specifically, we will use maximum likelihood estimation method, assuming that data are missing at random and conditional on observed information. This assumption is reasonable given that reasons for attrition will be well monitored in our study. We will model the growth process for sleep to see if there is a significant change. For maximum likelihood estimation of mixed effects models, we will use a latent variable modeling software. Exploratory correlational hypotheses will be tested by first plotting data and determining best fit linear model (fitting with the Kolmogorov-Smirnov algorithm and fit comparisons with Akaike's Information Criterion).

Given the nature of a feasibility study within the R21 framework, we will take into account both statistical significance (p-value) and clinical significance (effect size) in our inference, with more emphasis on the latter. Nonetheless, we calculated the sample size based on the primary outcome measure of the "falling asleep" dimension of the ASWS assuming that the magnitude of the treatment effect would be large. That is, we expect to detect a difference of 0.96 units (large clinical effect size, Cohen's d=0.80). Given that we do not have the detailed information necessary to conduct power calculations for mixed effects modeling, power was estimated based on simple mean comparison at post-treatment follow up assessment. We expect that the power will increase somewhat as we actually analyze the data using the mixed effects modeling by utilizing repeated measures and by properly handling missing data. We use the nominal 5% significance level (two-tailed). Based on this scenario, with 60 subjects (30 control, 30 treatment), estimated power is 0.86, which goes down to 0.73 if adjusted for the multiple outcomes we compare in Hypotheses 1 and 2 ($\alpha = 0.05/3 = 0.016$).

Title : Treating Sleep Disruption in Teens with Millisecond Light Exposure During Sleep
Approval Period: Draft

[redacted]

2. Study Procedures

a) Please SUMMARIZE the research procedures, screening through closeout, which the human subject will undergo. Refer to sections in the protocol attached in section 16, BUT do not copy the clinical protocol. Be clear on what is to be done for research and what is part of standard of care.

Students (n=60) in grades 9-12 who have symptoms consistent with a diagnosis of DSPD will be recruited for an at-home study of the effectiveness of flashes of light in alleviating sleep problems. All subjects will have an initial visit (~1.75 hours) in the company of a parent or guardian during which baseline questionnaires will be completed. A desired wake time will

be established by consensus between the adolescent and parent. Within seven days of this baseline visit, a technician will visit the subject in their home to install an automated flash device in the subject's bedroom. The device will be positioned near the subject's bed to optimize exposure to the flashes during sleep. For the next four weeks, the device will be set to deliver three hours of flashes beginning three hours before the target wake time. No experimental restrictions will be placed on the subjects' bed or wake times, though we will ask that the subject complete a daily sleep log. The timing of the light will be the same on both weekdays and weekends. At the end of each seven days of the study (i.e., Days 7, 14, 21, and 28), a technician will contact the subject and administer questionnaires over the telephone that concern sleep behavior. The technician will also answer any questions the subject or the parents might have. This phone call will be used to maintain contact with subjects during the study period and also be used to ascertain any untoward events. Within seven days of the end of the four-week flash exposure, subjects will be asked to return to the laboratory with the flash equipment and complete a follow-up set of questionnaires (~30 minutes).

Subjects will be scheduled to receive three hours of light during the three hours prior to target waketime, when the subject is anticipated to be asleep. Half of all consented subjects will be assigned to a control condition in which they will be exposed to a subthreshold light stimulus of one light flash every thirty minutes. The other half will receive one flash every 30 seconds. Each flash will be white light (xenon flash buln) of an illuminance of approximately 4,000 lux of ambient light (~200-600 lux at the cornea). All subjects will be told that they will receive light at night. We will also advise that if the parents/guardians of the subject are awake and see the flashes, they are not to reveal the condition to the subject. The light condition will be double-blind as the researcher analyzing the data will not know the conditions to which the subjects were exposed. During the weekly phone call, the technician will inquire as to whether subjects were awake during the time that the flashes were supposed to be administered and whether they saw anything. Subjects who report being awake during this time may be influenced by what they see (or do not see, as the case may be) and this information will, therefore, be used as a covariate in our analyses. We will use block randomization to assign subjects to conditions in groups of 10 by season (Fall, Winter, Spring).

In order to examine the effects of our intervention, we will administer a series of questionnaires at both Baseline and End of Treatment. The same questionnaires will be administered at both time points. These questionnaires assess sleep quality, chronotype, stress reactivity, daytime sleepiness, and depressive symptomatology. We will also administer questionnaires to parents concerning Attention Deficit Hyperactivity Disorder (ADHD) symptomatology and
sleep in the adolescent being studied. If a potential subject does not have a formal diagnosis of DSPD (see Inclusion Criteria, below), we will also administer Module 4 (Circadian Rhythm Sleep Disorders) of the Duke Structured Interview for Sleep Disorders, a validated structured interview that can reliably diagnose sleep disorders consistent with definitions described by the International Classification of Sleep Disorders (ICSD-2) and the Diagnostic and Statistical Manual of Mental Disorders (DSM-IV).

Sleep quality will be assessed using the Adolescent Sleep Wake Scale (ASWS), a well-validated, internally consistent measure of sleep quality in 12-17 year olds that measures five behavioral dimensions: going to bed, falling asleep, maintaining sleep, reinitiating sleep,
and returning to wakefulness.

Different dimensions of ADHD symptomatology will be measured with the SNAP-IV 26, a widely-used parent- or teacher-completed short-form of the original 90-item Swanson, Nolan, and Pelham (SNAP)-IV Questionnaire commonly used for assessment of ADHD
symptoms. A subscore for each of the three ADHD dimensions (inattention, hyperactivity/impulsivity, oppositional defiant disorder) will be computed. It has been hypothesized that a significant number of children and adolescents who have sleep

disruption are misdiagnosed as having ADHD. The DSM-IV-TR inattention criteria for ADHD have a significant overlap with symptoms of insufficient sleep and many children can exhibit paradoxical hyperactivity in response to sleepiness (e.g., such as occurs after taking antihistamines).

Depressive symptomatology will be measured with the Center for Epidemiologic Studies Depression Scale for Children (CES-DC), a well-validated, internally consistent measure designed for use in adolescents. There is a strong link between clinical depression and sleep disruption in adults and, to a lesser degree adolescents.

Chronotype is a measure of the degree of morning or evening preference ("lark" versus "owl") that we will assess using the Morningness-Eveningness Scale for Children (MESC). The MESC was initially validated in 11-12 year olds, but has been used in those aged 12-17 as well.
The relationship between the timing of sleep and circadian rhythms varies in extreme chronotypes. We hypothesize that MESC score will act as a moderator variable such that individuals with lower MESC scores (less morningness) will have greater improvements in sleep.

We will assess daytime sleepiness with the Cleveland Adolescent Sleepiness Questionnaire (CASQ), a well-validated, internally consistent measure of daytime sleepiness for adolescents. As daytime sleepiness is often directly attributable to quality and quantity of sleep, we will examine as an exploratory measure the hypothesis that our intervention will decrease daytime sleepiness and that such decreases will be associated with improvements in sleep.
Parent impression of sleep in the teen will be measured with the Child and Adolescent Sleep Checklist (CASC). This questionnaire has 24 questions from which an overall sleep disturbance score can be calculated, as well as subscores concerning bedtime problems, sleep breathing, parasomnias, and daytime problems.

To further explore domains relevant to ADHD, adolescents will complete The Brief Sensation Seeking Scale (BSSS). The BSSS is an 8-item self-report measure designed specifically for use with youth populations. Participants rate each item using a 5- point scale (1=strongly disagree; 5= strongly agree). Sensation-seeking and risk-taking propensity have been linked to both ADHD symptoms and evening preference in adolescent propulations.

As a measure of risk-taking propensity, participants will complete the Balloon Analogue Risk Task (BART). The BART involves pumping up a computer-simulated balloon, where each pump earns the participant money. However, if the balloon "pops" then the money is lost. At any point during the pumping period, a participant can stop and click the "collect money" box to put it into a permanent bank. Participants will be informed that they can win an Amazon gift card if they earn up to a specified amount of money. The BART has been widely used as a behavioral measure of risk-taking in adolescent samples.

As a measure of distress tolerance, participants will complete the Behavioral Indicator of Resiliency to Distress (BIRD). The BIRD is a computer task with three levels of increasing difficulty. Participants are asked to use the computer's mouse to click a box as soon as a green dot appears above it, and before the green dot moves to another location. If the box is clicked before the green dot moves, a simulated bird is released from a "cage" and a point is earned; if the box is not clicked in time, the bird remains in its cage and no point is earned. The green dot moves more quickly as the levels progress, and participants are given the option to 'Quit Game' (i.e. terminate the task) at any point in the third level. The BIRD has been used with adolescents in numerous studies as a measure of frustration tolerance.

Weekly questionnaires: In addition to the baseline and end of treatment questionnaires, we will also administer via telephone three of the questionnaires (ASWS, CASQ, CES-DC) at the end of each of the four study weeks. Administration of these questionnaires should take less than 10 minutes per week. We will use these to examine changes in sleep and daytime

sleepiness. We hypothesize that falling asleep and daytime sleepiness will progressively and significantly improve over each of the four weeks. The CES-DC and the CASQ will also be monitored for clinically significant changes in depressive symptomatology or sleepiness, respectively. An increase of more than 12 (CES-DC) or 11 (CASQ) from baseline will result in an immediate referral of the participant to Dr. Sullivan who will advise a clinically appropriate course of action.

Daily questionnaire: Every morning upon awakening, subjects will be asked to complete the Consensus Sleep Diary – Core, a brief log of basic information concerning the previous night of sleep that should take less than one minute to complete. Changes in sleep timing and self-rated quality of sleep will be examined as exploratory measures.

Daily wrist actigraphy: subjects will be asked to wear a wrist actigraph - a wrist watch-sized device (also looks like a wrist watch) that records three-dimensional movement. Data from this device will be useful in confirming the self-reported sleep times in the daily questionnaire.

[redacted]

Students in grades 9-12 (N=95) who have symptoms consistent with a diagnosis of DSPD will be recruited for an at-home study of the effectiveness of flashes of light combined with a brief behavioral treatment in alleviating sleep problems. All subjects will have an initial visit (~1.75 hours at either the participant's home or at an office at Stanford University) in the company of a parent or guardian during which baseline questionnaires will be completed. A desired wake time will be established by consensus between the adolescent and parent. Within seven days of this baseline visit, a technician will visit the subject in their home to install an automated flash device in the subject's bedroom. The device will be positioned near the subject's bed to optimize exposure to the flashes during sleep. For the next four weeks, the device will be set to deliver three hours of flashes beginning three hours before the target wake time. We will ask that subjects attempt to fall asleep at least one hour earlier than their current bed time. We will ask that the subject complete a daily sleep log and respond to a single daily (evening) question about their current alertness. The timing of the light will be the same on both weekdays and weekends.

After the light has been placed in the participants' bedroom, a subset of participants (N=35) will be invited to attend four weekly therapy sessions. Consistent with other brief behavioral treatments for sleep disturbances, the behavioral intervention will consist of four, 50-minute weekly, in-person sessions delivered by a licensed clinical psychologist. The behavioral includes five components: (1) Motivational Interviewing, a straightforward, non-directive discussion about attitudes towards behavior change, including the importance and likelihood of making sleep changes and ways in which sleep changes (i.e., increased sleep) may be consistent with the adolescent's values; (2) Education on the circadian system, the impact of light, and physiological processes that operate during sleep and wakefulness; (3) The Role of Sleep in domains relevant to adolescents, including athletic performance, physical appearance, weight loss/maintenance, and academics; (4) Sleep Hygiene (e.g. instructions to limit caffeine, exercise and light exposure in the evening) and Stimulus Control (e.g. instruction to reserve the bed for sleep only), both well-established insomnia treatment components designed to improve sleep and associated contextual cues; and (5) Activity Scheduling to create routines and anticipate obstacles (e.g. homework, socializing that may interfere with opportunity to sleep).

To evaluate whether the content of the behavioral intervention was administered at each session as planned (i.e., treatment fidelity), sessions will be audiotaped and an independent reviewer, a member of the study team, will randomly review a selection of therapy tapes to assess treatment fidelity. Audio files of sessions will be stored on Stanford's HIPAA-compliant School of Medicine cloud-based storage, and deleted from the digital audio recorder once transferred. After treatment fidelity has been established, all audio files will be destroyed. Audio will not be used for any other purpose other than for internal review of treatment fidelity.

During these behavioral intervention study visits, we will administer questionnaires that concern sleep behavior. We will also answer any questions the subject or the parents might have and ascertain any untoward events. Within seven days of the end of the four-week flash exposure and behavioral intervention visits, subjects will be asked to return to the laboratory with the flash equipment and complete a follow-up set of questionnaires (~30 minutes).

Subjects will be scheduled to receive three hours of light during the three hours prior to target waketime, when the subject is anticipated to be asleep. Half of all consented subjects will be assigned to a control condition in which they will be exposed to a subthreshold light stimulus of one light flash every thirty minutes. The other half will receive one flash every 30 seconds. Each flash will be white light (xenon flash bulb) of an illuminance of approximately 4,000 lux of ambient light (~200-600 lux at the cornea). All subjects will be told that they will receive light at night. We will also advise that if the parents/guardians of the subject are awake and see the flashes, they are not to reveal the condition to the subject. The light condition will be double-blind as the researcher analyzing the data will not know the conditions to which the subjects were exposed. During the weekly therapy visit, the therapist will inquire as to whether subjects were awake during the time that the flashes were supposed to be administered and whether they saw anything. Subjects who report being awake during this time may be influenced by what they see (or do not see, as the case may be) and this information will, therefore, be used as a covariate in our analyses. We will use block randomization to assign subjects to conditions in groups of 10 by season (Fall, Winter,

).

In order to examine the effects of our intervention, we will administer a series of questionnaires at both Baseline and End of Treatment. The same questionnaires will be administered at both time points. These questionnaires assess sleep quality, chronotype, stress reactivity, daytime sleepiness, and depressive symptomatology. We will also administer questionnaires to parents concerning Attention Deficit Hyperactivity Disorder (ADHD) symptomatology and sleep in the adolescent being studied. If a potential subject does not have a formal diagnosis of DSPD (see Inclusion Criteria, below), we will also administer Module 4 (Circadian Rhythm Sleep Disorders) of the Duke Structured Interview for Sleep Disorders, a validated structured interview that can reliably diagnose sleep disorders consistent with definitions described by the International Classification of Sleep Disorders (ICSD-2) and the Diagnostic and Statistical Manual of Mental Disorders (DSM-IV).

Sleep quality will be assessed using the Adolescent Sleep Wake Scale (ASWS), a well-validated, internally consistent measure of sleep quality in 12-17 year olds that measures five behavioral dimensions: going to bed, falling asleep, maintaining sleep, reinitiating sleep, and returning to wakefulness.

Different dimensions of ADHD symptomatology will be measured with the SNAP-IV 26, a widely-used parent- or teacher-completed short-form of the original 90-item Swanson, Nolan, and Pelham (SNAP)-IV Questionnaire commonly used for assessment of ADHD symptoms. A subscore for each of the three ADHD dimensions (inattention, hyperactivity/impulsivity, oppositional defiant disorder) will be computed. It has been hypothesized that a significant number of children and adolescents who have sleep disruption are misdiagnosed as having ADHD. The DSM-IV-TR inattention criteria for ADHD have a significant overlap with symptoms of insufficient sleep and many children can exhibit paradoxical hyperactivity in response to sleepiness (e.g., such as occurs after taking antihistamines).

Depressive symptomatology will be measured with the Center for Epidemiologic Studies Depression Scale for Children (CES-DC), a well-validated, internally consistent measure designed for use in adolescents. There is a strong link between clinical depression and sleep disruption in adults and, to a lesser degree adolescents.

Chronotype is a measure of the degree of morning or evening preference ("lark" versus "owl") that we will assess using the Morningness-Eveningness Scale for Children (MESC). The MESC was initially validated in 11-12 year olds, but has been used in those aged 12-17 as well. The relationship between the timing of sleep and circadian rhythms varies in extreme chronotypes. We hypothesize that MESC score will act as a moderator variable such that individuals with lower MESC scores (less morningness) will have greater improvements in sleep.

We will assess daytime sleepiness with the Cleveland Adolescent Sleepiness Questionnaire (CASQ), a well-validated, internally consistent measure of daytime sleepiness for adolescents. As daytime sleepiness is often directly attributable to quality and quantity of sleep, we will examine as an exploratory measure the hypothesis that our intervention will decrease daytime sleepiness and that such decreases will be associated with improvements in sleep.

Parent impression of sleep in the teen will be measured with the Child and Adolescent Sleep Checklist (CASC). This questionnaire has 24 questions from which an overall sleep disturbance score can be calculated, as well as subscores concerning bedtime problems, sleep breathing, parasomnias, and daytime problems.

Adolescent sleep-related behaviors will be explored using the Adolescent Sleep Habits Survey, a measure created by pediatric sleep researchers at Brown University. This questionnaire asks about schedule variability, pre-sleep behaviors and health behaviors (e.g. caffeine use) that might interfere with sleep.

To evaluate motivation for changing sleep-related behaviors, we will administer the Behavioral Intentions Questionnaire (BIQ). This 5-item instrument explores participant motivations to change in five key sleep-related areas, and has been used in prior school- and therapy-based interventions to improve adolescent sleep.

To further explore domains relevant to ADHD, adolescents will complete The Brief Sensation Seeking Scale (BSSS). The BSSS is an 8-item self-report measure designed specifically for use with youth populations. Participants rate each item using a 5- point scale (1=strongly disagree; 5= strongly agree). Sensation-seeking and risk-taking propensity have been linked to both ADHD symptoms and evening preference in adolescent populations.

As a measure of risk-taking propensity, participants will complete the Balloon Analogue Risk Task (BART). The BART involves pumping up a computer-simulated balloon, where each pump earns the participant money. However, if the balloon "pops" then the money is lost. At any point during the pumping period, a participant can stop and click the "collect money" box to put it into a permanent bank. Participants will be informed that they can win an Amazon gift card if they earn up to a specified amount of money. The BART has been widely used as a behavioral measure of risk-taking in adolescent samples.

As a measure of distress tolerance, participants will complete the Behavioral Indicator of Resiliency to Distress (BIRD). The BIRD is a computer task with three levels of increasing difficulty. Participants are asked to use the computer's mouse to click a box as soon as a green dot appears above it, and before the green dot moves to another location. If the box is clicked before the green dot moves, a simulated bird is released from a "cage" and a point is earned; if the box is not clicked in time, the bird remains in its cage and no point is earned. The green dot moves more quickly as the levels progress, and participants are given the option to 'Quit Game' (i.e. terminate the task) at any point in the third level. The BIRD has been used with adolescents in numerous studies as a measure of frustration tolerance.

Weekly questionnaires: In addition to the baseline and end of treatment questionnaires, we will also administer via telephone three of the questionnaires (ASWS, CASQ, CES-DC) at the end of each of the four study weeks. Administration of these questionnaires should take less than 10 minutes per week. We will use these to examine changes in sleep and daytime sleepiness. We hypothesize that falling asleep and daytime sleepiness will progressively and significantly improve over each of the four weeks. The CES-DC and the CASQ will also be monitored for clinically significant changes in depressive symptomatology or sleepiness, respectively. An increase of more than 12 (CES-DC) or 11 (CASQ) from baseline will result in an immediate referral of the participant to Dr. Sullivan who will advise a clinically appropriate course of action.

Daily questionnaire: Every morning upon awakening, subjects will be asked to complete the Consensus Sleep Diary – Core, a brief log of basic information concerning the previous night of sleep that should take less than one minute to complete. Changes in sleep timing and self-rated quality of sleep will be examined as exploratory measures.

Evening alertness question: Each evening, participants will receive a text or email asking: "How sleepy do you feel right now?" (in the subject line so it appears bold in the text message) then "Please rate your response on a scale of 1-7, 1='very little' and 7='very much'" (in the content area)

Daily wrist actigraphy: subjects will be asked to wear a wrist actigraph - a wrist watch-sized device (also looks like a wrist watch) that records three-dimensional movement. Data from this device will be useful in confirming the self-reported sleep times in the daily questionnaire.

Pilot Evaluation: Prior to combining our behavioral intervention and light intervention, we wish to run N=10 adolescents through all aspects of the behavioral intervention only. We will complete this pilot evaluation during summer 2015, before the school year begins. Our pilot evaluation will be identical to the full study protocol (i.e. baseline visit, five weeks of data collection, four therapy visits, post-treatment visit)

with the exception that no light will be administered at any point during the pilot phase of our treatment. Participants will also be asked at the final study visit to provide feedback on the intervention and discuss aspects of the program they felt were more or less helpful.

[redacted]

[redacted]

[redacted]

[redacted]

[redacted]

[redacted]

[redacted]

[redacted]

[redacted]

[redacted]

[redacted]

[redacted]

[redacted]

[redacted]

[redacted]

[redacted]
