# Supplementary material for: Effect of Light Flashes vs Sham Therapy During Sleep With Adjunct Cognitive Behavioral Therapy on Sleep Quality Among Adolescents: A Randomized Clinical Trial
Source: JAMA Netw Open. 2019 Sep 25;2(9):e1911944. doi: 10.1001/jamanetworkopen.2019.11944 (PMC6763980; doi:10.1001/jamanetworkopen.2019.11944)
Supplement: Supplement 2. — Data Sharing Statement [file jamanetwopen-2-e1911944-s002.pdf]

Data Sharing Statement

Kaplan. Effect of Light Flashes vs Sham Therapy During Sleep With Adjunct Cognitive Behavioral Therapy on Sleep Quality Among Adolescents. *JAMA Netw Open*. Published September 25, 2019.
10.1001/jamanetworkopen.2019.11944

Data
Data available: Yes
Data types: Deidentified participant data
How to access data: All data are contained within the manuscript. Additional clarification can be obtained from the corresponding author, jzeitzer@stanford.edu
When available: With publication

Supporting Documents
Document types: None

Additional Information
Who can access the data: anyone requesting the data
Types of analyses: for any purpose
Mechanisms of data availability: with a signed data access agreement approved by Stanford University
